# Countermovement Jump Training Is More Effective Than Drop Jump Training in Enhancing Jump Height in Non-professional Female Volleyball Players

**DOI:** 10.3389/fphys.2020.00231

**Published:** 2020-03-17

**Authors:** Jan Ruffieux, Michael Wälchli, Kyung-Min Kim, Wolfgang Taube

**Affiliations:** ^1^Department of Neurosciences and Movement Sciences, Université de Fribourg, Fribourg, Switzerland; ^2^Department of Kinesiology and Sport Sciences, University of Miami, Coral Gables, FL, United States

**Keywords:** stretch-shortening cycle, CMJ, DJ, jump performance, volleyball

## Abstract

The aim of the present study was to compare the effects of countermovement jump (CMJ) and drop jump (DJ) training on the volleyball-specific jumping ability of non-professional female volleyball players. For that purpose, 26 female volleyball players (15–32 years) were assigned to either a CMJ (20.4 ± 3.1 years, 171.0 ± 3.0 cm) or a DJ training group (22.0 ± 4.4 years, 168.2 ± 5.0 cm), which performed a six-week jump training (two sessions per week, 60 jumps per session). Each group performed 20% of the jumps in the jump type of the other group in order to minimize the influence of enhanced motor coordination on the differences between groups regarding the improvements of jump performance. Before and after the training, jump height was assessed in four jump types, including the trained and volleyball-specific jump types. Although both training forms substantially improved jump height, the CMJ training was significantly more effective in all jump types (17 vs. 7% on average; *p* < 0.001). This suggests that, at least for non-professional female volleyball players and a training duration of six weeks, training with a high percentage of CMJs is more effective than one with a high percentage of DJs. We hypothesize that this might be related to the slower stretch-shortening cycle during CMJs, which seems to be more specific for these players and tasks. These findings should support volleyball coaches in designing optimal jump trainings.

## Introduction

In volleyball, a player’s maximal height above the net is a key determinant for successful attacking and blocking, and thus, for performance. The critical factors for this maximal height are anthropometric characteristics (body height and arm length) and vertical jumping ability. While the former cannot be modified, an athlete’s jumping ability can be significantly improved through training. Volleyball coaches, therefore, seek for the most effective and most efficient exercises to improve their players’ jumping ability.

The most common jump types in volleyball, i.e., for attacking and for blocking, can be classified as countermovement jumps (CMJs). More precisely, the block jump often resembles a “shortened version” of a CMJ due to time constraints, which prevent the players from performing a classic CMJ (Sattler et al., 2012). The attack jump on the other hand, which is performed with a run-up, can be regarded as a combination of a drop jump (DJ) and a CMJ (Sattler et al., 2012). CMJs and DJs are stretch-shortening cycle (SSC) movements, which involve a high-intensity eccentric contraction immediately before a rapid concentric contraction (Komi and Bosco, 1978; Van Hooren and Zolotarjova, 2017). Thus, in order to maximize performance in these jumps, it is important to quickly switch from yielding work to overcoming work and to rapidly develop maximal forces during the concentric phase (Bosco et al., 1981; Bobbert, 1990). Therefore, exercises aimed at improving jump performance in volleyball must target these reactive and explosive abilities of the neuromuscular system.

The most obvious training method to improve jump height is to perform jumps. This method is also referred to as plyometric training or plyometrics. Compared to other training methods often applied for improving jump performance, such as resistance training or weight lifting, jumps can be practiced everywhere and without any equipment. Furthermore, jumps represent the most specific training method. Not surprisingly, CMJ training has been shown to improve jump performance (Holcomb et al., 1996; Gehri et al., 1998). Another exercise for improving jumping ability, which has often been advised and which is often used by coaches, are DJs. DJs involve jumping or dropping from a raised platform and performing a vertical jump immediately after landing. The development of this method is ascribed to the Russian athletics coach Yuri Verhoshanski (Bobbert, 1990). For his “depth jumps,” Verhoshanski used drop heights of 0.75 and 1.1 m (Verhoshanski, 1967). For him, this so-called “shock” method should be incorporated only “in the later stages of a program over many years of specialized strength preparation” (Bobbert, 1990, p. 10). Because of the success of Verhoshanski’s athletes, coaches and scientists around the world adopted the idea of depth or drop jumping as an effective training method and started to develop it. Today, DJs – mainly with lower drop heights of around 20–50 cm – are integrated in strength and conditioning programs by coaches on all levels.

It has been suggested that in order to enhance jump performance, the capacity of individual muscles to release energy (i.e., power output) must be increased (Bobbert, 1990). According to Bobbert (1990), this can be achieved with exercises that come as close as possible to the target exercise (in our case volleyball jumps) with regard to the characteristics of the movement (specificity) but during which the muscles produce a greater mechanical output (larger forces and power output) than during the target exercises (so-called training overload; Bobbert, 1990). The mechanical output of a muscle during a concentric contraction can be enhanced by prestretch (potentiation; Bosco et al., 1981; Bobbert, 1990). This potentiation effect depends on the speed of prestretch and the delay between the prestretch and the concentric phase (Bosco et al., 1981). The increased negative speed during a DJ compared to a CMJ increases the speed of prestretch of the knee extensors and the plantar flexors and decreases the delay between the prestretch and the concentric phase (Bobbert et al., 1987), leading to a greater mechanical output during the push-off phase, which is believed to stimulate a more effective utilization of the SSC (Bobbert, 1990; Moran and Wallace, 2007). Thus, DJs seem to meet these requirements of being specific and inducing training overload. In line with this, numerous studies have shown that DJ training can significantly improve vertical jumping ability (for reviews, see Bobbert, 1990; Markovic, 2007).

Very few studies, however, have compared the effect of DJ to that of CMJ training on CMJ or volleyball-specific jump performance and they suggest that the two methods are equally effective (Clutch et al., 1983; Holcomb et al., 1996; Gehri et al., 1998). Improvements in jump height can be the consequence of both improvements in the capacity of muscles to release energy (peripheral mechanisms) and intra- and intermuscular coordination (central mechanisms; Bobbert, 1990). It could be argued that the similar effects of the two training methods on CMJ performance in previous studies (which compared pure CMJ training to pure DJ training) could be explained by a greater effect of the DJ training on the capacity of muscles to release energy (see above), which was compensated for by more specific neural adaptations in the CMJ groups. It has been shown that different forms of strength training lead to task-specific neural adaptations (Giboin et al., 2018). Therefore, the aim of the present study was to compare the effects of CMJ and DJ training on (volleyball-specific) jump performance while trying to minimize the influence of enhanced motor coordination on the differences between groups regarding the improvements of jump performance. To this end, we compared a CMJ with a DJ training group with each group completing 20% of the jumps in the other form (i.e., the CMJ training group performed 80% CMJs and 20% DJs while the DJ training group performed 80% DJs and 20% CMJs). We expected that the 80% DJs would allow the DJ group to benefit to a great extent from the greater mechanical output during this jump type, while the 20% CMJs would be sufficient to induce task-specific motor coordination improvements in the CMJ. Therefore, we hypothesized that the proposed DJ training would be more effective in improving overall vertical jumping ability.

## Materials and Methods

### Participants

Thirty-three female volleyball players (15–32 years) of three different teams participated in the study. All teams play on a regional level and practice at least two times per week. All players were experienced with jump and plyometric training (4–15 years) but had no history of a long-term specialized jump and resistance training. The participants were assigned to either a CMJ or a DJ training group, which were matched for jump height at the pre-test, age, and team affiliation. Seven participants had to be excluded from the study due to injuries unrelated to the intervention (*n* = 2) or because they attended less than 80% of the training sessions (*n* = 5). Thus, 13 participants were included in the analysis for the CMJ (20.4 ± 3.1 years, 171.0 ± 3.0 cm) and 13 participants for the DJ training group (22.0 ± 4.4 years, 168.2 ± 5.0 cm). Written informed consent was obtained from all participants, and from a parent for underage participants, prior to participation. The study was approved by the local ethics committee and was in accordance with the latest version of the Declaration of Helsinki.

### Study Design

This training study consisted of a six-week jump training. In pre- and post-measurements, jump performance was assessed in four different jump types. During the training, the participants mainly performed either CMJs or DJs, according to their group. The jump types as well as the measurements and the training are described in detail below.

### Measurements

Before and after training, jump performance was assessed in four different jump types: (a) CMJ with the hands akimbo, (b) CMJ with arm swing (which is similar to a block jump in volleyball), (c) CMJ with run-up and arm swing (identical to an attack jump in volleyball), and (d) DJ with arm swing with a drop height of 37 cm. Jump heights were calculated from flight times, which we measured with an OptoGait system (Microgate Srl, Bolzano, Italy). A very high degree of validity and reliability has been demonstrated for both the tested jump types and the measuring instrument used (Carroll et al., 2019; Glatthorn et al., 2011; Sattler et al., 2012). In addition, ground contact times were recorded for the DJs. Five jumps were recorded in each jump type, resulting in a total number of 20 jumps. The low number of jumps should prevent effects of fatigue. The order of the jump types was randomized between participants. For all jumps, the participants were instructed to jump as high as possible. No instructions were given regarding the range of motion or ground contact time. They received feedback about their jump height after each jump. Before each measurement session, the participants performed a standardized specific warm-up, which also included the four jump types assessed during the measurements.

### Training

The training lasted six weeks, with two sessions per week. This corresponds to a typical volume of a pre-season athletic training during summer for players of this level. The jump training was performed at the beginning of the regular training sessions of the teams and was led by an experimenter. The actual jump training was always preceded by the same standardized warm-up that was conducted before the measurements. Each participant performed 60 jumps per session, which were grouped in blocks of three jumps with 3–5 s rest in between jumps and 30 s in between blocks. Five blocks constituted one series and one session comprised four series with 2 min rest between series. According to their group, the participants performed either CMJs (with arm swing but without run-up) or DJs (with arm swing, drop height of 37 cm).

However, each group performed one block per series (i.e., 20% of the jumps) in the jump type of the other group. With this we wanted to reduce the influence of task-specific improvements in motor coordination on the differences between groups regarding the improvements of jump performance. The participants were encouraged to perform each jump maximally. In order to maximize their motivation, the jump height was fed back to the participants in one of six jumps on average.

### Statistical Analyses

For each participant, jump type, and time point, the best of the five trials was used for statistical analysis. We performed a linear mixed effects analysis of the effect of the two training modalities on jump height. As fixed effects, we entered group, time point, and jump type and all interaction terms into the model. As random effects, we had intercepts for subjects and by-subject random slopes for the effects of group and time point. A similar analysis was performed on the ground contact times during the DJs with group, time point, and the interaction term as fixed effects and intercepts for subjects as random effects. Visual inspection of residual plots did not reveal any obvious violations of the homoscedasticity or normality assumptions. The significance of the fixed effects was tested using Kenward–Roger’s *F*-test with an alpha level of 0.05. The analyses were performed using R (R Core Team, 2018) and the *lmerTest* package (Kuznetsova et al., 2017).

## Results

**FIGURE 1:**
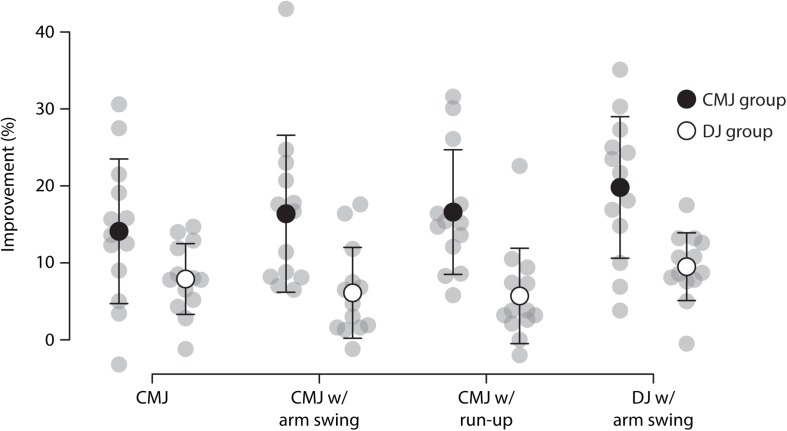
Percent improvement for the countermovement jump (CMJ; filled circles) and the drop jump (DJ; open circles) training groups in the four jump types. Gray circles represent the individual participants. Error bars represent the standard deviation.

**FIGURE 2:**
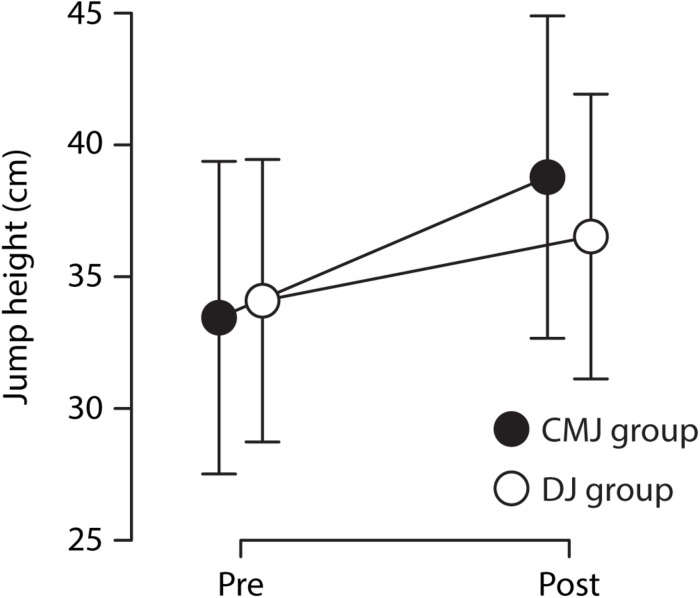
Mean jump heights (over all jump types) for the countermovement jump (CMJ) and the drop jump (DJ) training groups before (Pre) and after (Post) training. Statistical analysis showed that the improvement was significantly greater in the CMJ training group (*p* < 0.001). Error bars represent the standard deviation.

**FIGURE 3:**
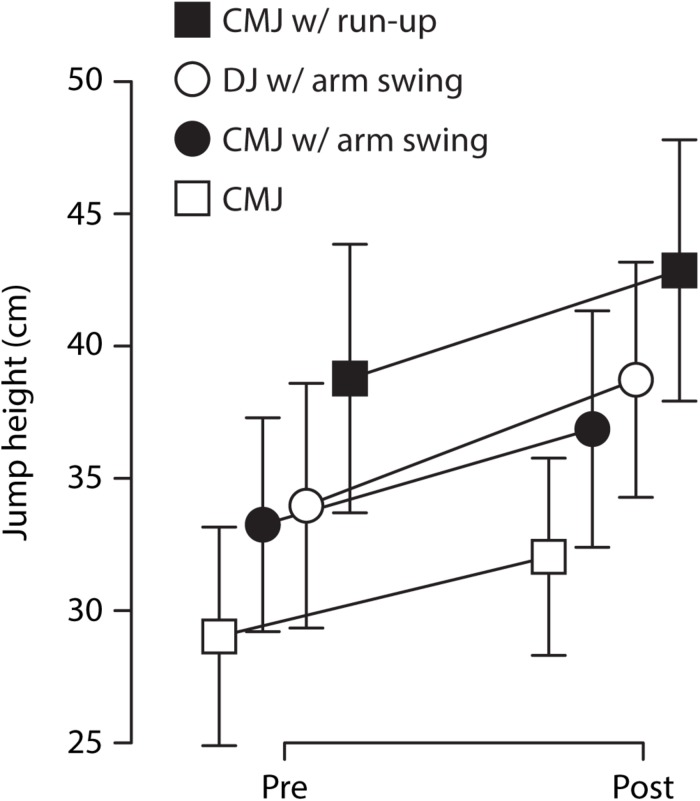
Mean jump heights (over both groups) in the four jump types before (Pre) and after (Post) training. Statistical analysis showed that jump height (*p* < 0.001) and improvements (*p* = 0.002) differed between jump types, with the greatest jump heights in the countermovement jump (CMJ) with run-up and the greatest improvements in the drop jump (DJ). Circles represent the jump types that were practiced. Error bars represent the standard deviation.

Table 1 shows the group mean jump heights before and after training in the four jump types. The percent improvements of the two groups in the four jump types are illustrated in Figure 1. Averaged over the four jump types, the CMJ training group improved jump height by 16.7 ± 9.2%, the DJ training group by 7.3 ± 5.4%. The statistical analysis showed that this group difference was significant, as indicated by the significant interaction of group and time point, *F*(1,24) = 22.05, *p* < 0.001, $\eta_{p}^{2}=0.48$ (see Figure 2). The three-way interaction (i.e., group × time point × jump type) was not significant (*p* = 0.064), indicating that the CMJ training group improved more than the DJ training group in all jump types. The effect of time point, *F*(1,24) = 152.35, *p* < 0.001, $\eta_{p}^{2}=0.86$, indicates significant overall training improvements. The analysis further revealed an effect of jump type, *F*(3,22) = 209.04, *p* < 0.001, $\eta_{p}^{2}=0.97$, and an interaction of jump type and time point, *F*(3,72) = 5.33, *p* = 0.002, $\eta_{p}^{2}=0.18$, suggesting that absolute jump heights and improvements were different between jump types, with the greatest jump heights in the CMJ with run-up and the greatest improvements in the DJ (see Figure 3).

**TABLE 1:** Jump heights (in cm) before (Pre) and after (Post) training in the four jump types for the countermovement jump (CMJ) and the drop jump (DJ) training groups.

	**CMJ training group**	**DJ training group**
**CMJ**		
Pre	28.8 ± 4.5	29.2 ± 3.8
Post	32.7 ± 4.0	31.4 ± 3.5
**CMJ w/arm swing**		
Pre	32.7 ± 4.7	33.8 ± 3.4
Post	37.8 ± 4.1	35.9 ± 4.8
**CMJ w/run-up**		
Pre	38.7 ± 5.2	38.8 ± 5.2
Post	44.9 ± 4.5	40.9 ± 4.7
**DJ w/arm swing**		
Pre	33.4 ± 5.1	34.6 ± 4.3
Post	39.7 ± 4.7	37.8 ± 4.1

*Values represent the mean ± standard deviation.*

After removal of one outlier in the CMJ group, the ground contact times during the DJs were 339 ± 71 ms before and 380 ± 79 ms after the training in the CMJ group and 317 ± 73 ms and 318 ± 48 ms, respectively, in the DJ group. The statistical analysis showed no significant effect of group or time point (all *p* > 0.082).

## Discussion

The six-week jump training led to a substantial increase in jump height in both groups. However, against our hypothesis, training with 80% CMJs led to significantly greater improvements (16.7% on average) than training with 80% DJs (7.3% on average), in all jump types. The largest group differences were found for the CMJ with run-up and the CMJ with arm swing, which can be compared with an attack or block jump in volleyball, respectively. In these jumps, the CMJ group was able to increase their maximum jump height by 6.1 and 5.1 cm, respectively, – a difference that can have a big impact on the performance in the game. In contrast, the DJ group increased jump height in these jumps by 2.1 cm. Thus, training has led to major, volleyball-specific improvements, especially in the CMJ group.

### CMJ vs. DJ Training

The question then arises as to why CMJ training was so much more effective than DJ training in the present study. As mentioned in the introduction, jump performance can be improved by both peripheral and central adaptations. Although the present study design does not allow a distinction between the two, the fact that the CMJ group was able to make greater progress than the DJ group even in the DJs suggests, at first glance, that the group differences are not (only) due to greater central but (also) to greater peripheral adaptations. However, if we look at the ground contact times during the DJs, we see that they were rather long (>300 ms) in both groups and tended to become even longer through training in the CMJ group (although not statistically significant). The players obviously performed so-called “countermovement” DJs and not “bounce” DJs (Marshall and Moran, 2013). Thus, it is conceivable that the CMJ group was able to improve DJ performance more because they learned to extend ground contact time in order to develop greater impulse. A similar observation was made in a study comparing the effect of a DJ training with a fixed drop height of 30 cm to that of a training with varying (greater) drop heights (30, 50, and 75 cm; Taube et al., 2012). Training with the greater drop heights resulted in longer ground contact times combined with an increased DJ height, while training with a drop height of 30 cm reduced ground contact time without a significant change in jump height (Taube et al., 2012). From this point of view, the greater increase in performance of the CMJ group in the DJ could, nevertheless, be attributed, to some extent, to central adaptations. Considering the long ground contact times during the DJs, we are talking about rather slow SSCs here, which are even longer for the CMJ forms. From this perspective, the CMJ was closer to the target forms (both CMJ forms and DJ) for the population of this study and the CMJ training thus led to more specific central adaptations. This would be in line with the training specificity principle (Giboin et al., 2018).

A second possible explanation for the different progress of the two groups concerns muscular activation. Since the participants were more familiar with the CMJ, it is conceivable that they had deficits in motor coordination in the DJ compared to the CMJ at the beginning of the training. The fact that both groups improved most in the DJ also indicates this. This could have led to a greater activation deficit during the DJs than during the CMJs. Thus, a larger percentage of muscle volume would have been active and loaded in the CMJ group, at least during the first weeks of training. This reasoning would argue for larger peripheral adaptations in the CMJ group. In most previous studies that compared DJ to CMJ training, training interventions were usually longer (Bobbert, 1990; Markovic, 2007), which could have compensated for differences in the activation deficit at the beginning of training. This could explain the differences between our results and those of previous studies, which could not find any differences in the training effect between CMJ and DJ training. However, this is speculative and cannot be answered by the present study as no recordings of muscle activity were made.

### Limitations

The above considerations lead us to a first limitation of this study, the target population. The participants of this study had some experience with jump training, but were non-professional players with no history of a long-term specialized jump and resistance training. It is conceivable that players with a higher technical and physical starting level could benefit more from DJ training than the sample of this study. Furthermore, we cannot say to what extent our findings can be transferred to male players.

A second point that needs to be discussed is the training protocol. Both groups performed 20% of the jumps in the other jump type. We chose this design in order to minimize the influence of enhanced motor coordination on the differences between groups regarding the improvements of jump performance. Although, from our point of view, this design is a strength of this study, the present data does not allow us to say whether a training regimen with CMJs only or with a higher percentage of DJs would be even more beneficial. Despite the relatively low percentage of 20% DJs in the CMJ group, we have to consider the possibility that these DJs were critical to training success, at least for performance in the DJ. Nevertheless, the findings suggest that a training regimen with a high percentage of CMJs is more effective than one with a high percentage of DJs. Already Verhoshanski did not suggest performing DJs exclusively but rather that drop jumping is just one of the exercises incorporated in a training program (Verhoshanski, 1967). Our results suggest that an optimal percentage of DJs could be rather low for volleyball players of this level. Furthermore, as mentioned above, the design does not allow a distinction to be made between peripheral and central adaptations.

A last point we would like to mention is the drop height. The drop height of 37 cm we used is within the range that is commonly used for DJ training. However, we cannot exclude the possibility that this drop height was not optimal for each participant and that a different height would have led to different adaptations. Following the discussion above, one can imagine that a greater drop height would have led to longer ground contact times and thus to greater improvements in the DJ group. Thus, further studies are needed in order to refine and extend our findings.

## Conclusion

The aim of the present study was to compare the effects of CMJ and DJ training on the volleyball-specific jumping ability of non-professional female volleyball players. Although both training forms substantially improved jump height, the CMJ training was significantly more effective. This suggests that, at least for non-professional female volleyball players and a training duration of six weeks, training with a high percentage of CMJs is more effective than one with a high percentage of DJs. We hypothesize that this might be related to the slower SSC during CMJs, which seems to be more specific for these players and tasks. These findings should support volleyball coaches in designing optimal jump trainings.

## Data Availability Statement

The raw data supporting the conclusions of this article will be made available by the authors, without undue reservation, to any qualified researcher.

## Ethics Statement

The studies involving human participants were reviewed and approved by Commission d’éthique de recherche du Canton de Fribourg. Written informed consent to participate in this study was provided by the participants’ legal guardian/next of kin.

## Author Contributions

All authors contributed to the conceptualization and the design of the study, critically revised the work for important intellectual content, and approved the final manuscript. JR performed the experiments, analyzed the data, and prepared the manuscript.

## Conflict of Interest

The authors declare that the research was conducted in the absence of any commercial or financial relationships that could be construed as a potential conflict of interest.
